# ESRD-associated immune phenotype depends on dialysis modality and iron status: clinical implications

**DOI:** 10.1186/s12979-018-0121-z

**Published:** 2018-07-17

**Authors:** Didier Ducloux, Mathieu Legendre, Jamal Bamoulid, Jean-Michel Rebibou, Philippe Saas, Cécile Courivaud, Thomas Crepin

**Affiliations:** 1 INSERM, UMR1098, Federation Hospitalo-Universitaire, INCREASE, Besançon, France; 2 Univ. Bourgogne-Franche-Comté, Faculté de Médecine et de Pharmacie, LabEx LipSTIC, Besançon, France; 3 Univ. Bourgogne-Franche-Comté, Faculté de Médecine et de Pharmacie, LabEx LipSTIC, Dijon, France; 4 CHU Besançon, Department of Nephrology, Dialysis, and Renal Transplantation, Besançon, France; 5 CHU Dijon, Department of Nephrology, Dialysis, and Renal Transplantation, Dijon, France; 6 EFS Bourgogne Franche-Comté, Plateforme de Biomonitoring, INSERM CIC 1431/UMR1098, Besançon, France

**Keywords:** Immune senescence, Dialysis, Inflammation, Iron overload, Acute rejection

## Abstract

**Background:**

End-stage renal disease (ESRD) causes premature ageing of the immune system. However, it is not known whether hemodialysis (HD) and peritoneal dialysis (PD) similarly affect the T cell system.

**Methods:**

The aim of our study was to analyse whether dialysis modality may mitigate ESRD-induced immune senescence. We explored a large population of patients (675 ESRD patients) and both confirmed and refined the results in a second cohort (84 patients).

**Results:**

HD patients exhibited higher inflammatory monocytes counts (44/mm^3^ (1–520) vs 36/mm^3^ (1–161); *p* = 0.005). Patients on HD also had higher frequency of CD8 T cells (24% (7–61) vs 22% (8–42); *p* = 0.003) and reduced CD4/CD8 ratio. Such results were confirmed in the second cohort. Moreover, both CD4 + CD57 + CD28- (3.25% (0–38.2) vs 1.05% (0–28.5); *p* = 0.068) and CD8 + CD57 + CD28- (38.5% (3.6–76.8) vs 26.1 (2.1–46.9); *p* = 0.039) T cells frequencies were increased in HD patients. Telomere length did not differ according to dialysis modality, but was inversely related to ferritin levels (*r* = − 0.33; p = 0.003). There was a trend towards higher telomerase activity in PD patients (11 ± 13 vs 6 ± 11; *p* = 0.053). Thymic function was not different in PD and HD patients. Patients on PD before transplantation had a higher risk of acute rejection after kidney transplantation (HR, 1.61; 95%CI, 1.02 to 2.56; *p* = 0.041).

**Conclusions:**

More pronounced inflammation with hemodialysis may induce premature aging of the immune system. This observation correlates with a lower risk of acute kidney rejection in patients previously on HD. Clinical consequences in patients maintained on dialysis should be determined.

**Trial registration:**

Trial registration: NCT02843867, registered July 8, 2016.

## Background

End-stage renal disease patients are more prone to infection [1], have a greater risk of virus-related cancer [2], and poorly respond to vaccination [3]. These comorbidities are at least in part related to a premature aging of the immune system. Accordingly, concordant studies reported accelerated thymus attrition, accumulation of terminally differentiated activated memory T cells (TEMRA), and reduction in telomere length in ESRD patients [4, 5]. Although the underlying mechanisms are not fully understood, chronic low-grade inflammation, oxidative stress, and epigenetics modifications have been implicated in immune senescence associated with loss of renal function [6, 7]. However, CMV infection also plays a major role in ESRD-induced immunological aging [4].

The uremia-associated immune dysregulation is amplified after the start of renal replacement therapy [8]. However, it is not known whether hemodialysis and peritoneal dialysis similarly affect the T cell system.

The aim of our study was to analyze whether dialysis modality may mitigate ESRD-induced immune senescence. We first explored a large population of patients before transplantation. This cohort was initially designed to define pre-transplant immune profile and subsequent post-transplant clinical outcomes. Main lymphocytes subsets (Naïve and memory CD4+ T cells, CD8+ T cells, recent thymic emigrants, B cells, and NK cells) and monocytes were analysed. The results were confirmed and refined in a second cohort designed to explore uremia-related immune senescence. More specifically, thymic function (T cell receptor TREC), replicative senescence (CD4 + CD28-CD57+ and CD8 + CD28-CD57+ T cells (TEMRA)), and telomere length were assessed. Clinical outcomes were analyzed.

## Materials and methods

### Patients and methods

#### Exploratory cohort

Research has been conducted in the 833 first consecutive RTR from the ORLY-EST study [9]. Briefly, ORLY-EST is an observational prospective study including incident renal transplant recipients (RTR) in seven French transplant centers (Besançon, Clermont-Ferrand, Dijon, Kremlin-Bicêtre, Nancy, Reims, Strasbourg). The main objective of this study is to describe interactions between immune status and post-transplant atherosclerosis. For each patient, blood samples were collected at time of transplantation and one year after. Sample collection was performed after regulatory approval by the French ministry of health (agreement number # DC-2008-713, June 11th 2009). The ethic committee of Franche-Comté study has approved the study (2008). Patients enrolled in the ORLY-EST study gave their written informed consent. Clinical data were prospectively collected.

Among 833 patients, 747 received a first transplant. Seventy-two patients (9.6%) had never been dialyzed. 675 patients (81%) were included in this cohort. One hundred and thirty eight patients were on PD (20.4%) and 537 on HD.

Calcineurin inhibitors and Mycophenolate Mofetil were widely used as immunosuppressive regimen.

Cytomegalovirus (CMV) prophylaxis was given according to each center practice. Almost all CMV-exposed patients received valganciclovir for 3 months. All CMV-naïve patients having received a CMV positive kidney received valganciclovir for 3 or 6 months. All patients received Pneumocystis antimicrobial prophylaxis with trimethoprim-sulfamethoxazole for at least 6 months.

#### Cognitive cohort

Research has been conducted in the 84 patients under dialysis from the IRIS study [5]. Briefly, two hundred and twenty-two patients from the Nephrology department of the University Hospital of Besançon have been prospectively included between September 1st 2013 and August 1st 2016. Patients were split in 3 groups according to CKD stage: group 1 with normal renal function (estimated Glomerular Filtration Rate (eGFR) > 60 ml/min/1.73m^2^, Modification of Diet in Renal Disease (MDRD) study equation or creatininemia < 120 μmol/l, *n* = 85), group 2 with severe chronic kidney disease stage IV (eGFR 15–30 ml/min/1.73m^2^, MDRD, *n* = 53), and group 3 with ESRD on dialysis (hemodialysis [HD]: *n* = 47 and peritoneal dialysis [PD]: *n* = 37, arbitrary eGFR at 10 ml/min/1.73m^2^). Exclusion criteria were history of cancer, viral infections (HBV, HCV and HIV viruses), past history of transplantation, immunosuppressive treatments or a recent infectious episode (< 3 months). Clinical parameters including: body mass index, diabetes mellitus (type 1 or 2), chronic heart failure diagnosed by a cardiologist, history of cardiovascular events (myocardial ischemia, stroke, peripheral arterial disease, and carotid endarterectomy), active tobacco defined by consumption of at least 1 cigarette per day, and statin treatment were recorded. Dialysis patients were included at least 6 months after the onset of renal replacement therapy. Dose and duration of dialysis were also recorded. All patients were previously informed and gave their consent. The study was approved by the ethic committee (Approval 13/686) and registered in clinicaltrials.gov (NCT02116270).

### T and B cell immunophenotypic analysis

Absolute numbers of CD4^+^ and CD8^+^ T cells were determined on fresh samples by a single platform flow cytometry approach using TetraCXP® method, Flow-Count® fluorospheres and FC500 cytometer (Beckman Coulter, Villepinte, France) according to manufacturer’s recommendations. PBMCs were isolated by density gradient centrifugation (Pancoll, Pan-Biotech GmBH Aidenbach, Germany) and cryopreserved. After thawing, PBMCs were washed twice in RPMI 1640 + GlutaMAX™-I medium (Invitrogen, Cergy-Pontoise, France) containing 10% fetal calf serum (Invitrogen), thereafter referred as complete medium. Cells were stained with the following conjugated antibodies directed against: CD3, CD4, CD8, C25, CD28, CD31, CD45RA, CD45RO, CD57, CD16, CD19, CD56. To detect intracellular FoxP3, surface staining PBMCs were processed using fixation buffer and permeabilization buffer (BD Biosciences, Le Pont de Claix, France) and incubated with the anti-FoxP3 antibody. Cell debris and doublets were excluded on the basis of side versus forward scatter. Cells were analyzed on a FACS CANTO II cytometer (BD Biosciences) using FACS Diva (BD Biosciences) software.

Recent thymic emigrants (RTE) were defined as CD45RA^+^CD31^+^CD4^+^ T cells [10]. Data were analyzed by considering the percentage of RTE among CD4^+^ T cells (RTE frequency or RTE%) and the absolute numbers of circulating RTE/mm^3^. Naive CD4^+^ and CD8^+^ T cells was defined as CD45RA^+^CD28^+^, and terminally differentiated CD4^+^ and CD8^+^ T cells was defined as CD57^+^CD28^−^.

Pro-inflammatory monocytes were stained on fresh samples with the following conjugated antibodies directed against CD45 (APC, Pharmingen), CD14 (ECD, Immunotech), CD16 (PC-7, Immunotech), HLA-DR (FITC, Pharmingen), CD86 (PE, Immunotech) according to the manufacturer’s recommendations.

Regulatory T cells (Treg) were defined as CD4 + CD25 + Foxp3+. This T cell population was analysed in a subset of patients from the exploratory cohort (*n* = 60 with the same proportion of patient on HD and PD).

### Relative telomere length (RTL)

DNA was extracted from isolated PBMC according to manufacturer’s instructions (QIAMP DNA Blood mini kit reference 51,106, Qiagen, Courtaboeuf, France). A Nanodrop ND-1000 spectrophotometer (Labtech, Palaiseau, France) was used to quantify genomic DNA. Genomic DNA was stored in TE buffer (10 mM Tris-HCl, 0.1 mM EDTA, pH 7.5) at 4 °C at a concentration of 10 ng/μL. DNA samples were diluted into pure water before starting real-time quantitative multiplex PCR runs described in supplementary data Thermocycler was CFX96 Real Time System (Bio-Rad) with Bio-Rad CFX Manager software to generate standard curve and calculate T/S ratio. Two T/S results were obtained for each sample, and the final reported result for one sample in a given run is the average of the 2 T/S values. Average T/S is expected to be proportional to the average telomere length per cell.

### Relative telomerase activity (RTA) measurement

Telomerase activity of T cells was examined using the TeloTAGGG Telomerase PCR ELISA PLUS kit (Applied, Roche Diagnostics, France) according to the manufacturer’s instructions and as described previously [11].

### Outcomes

#### Exploratory cohort

##### Acute rejection

Acute rejection was considered in the presence of serum creatinine elevation. Only biopsy-proven acute rejections were considered. Acute rejection was defined according to the Banff classification. Only cellular acute rejections were taken into account.

##### Infections

Methods to assess infectious complications have been previously described [11].

Briefly, diagnosis of severe bacterial infections required bacterial infection-related hospitalization. SBI was considered only if infection was the primary diagnosis for hospitalization.

All opportunistic infections (pneumocystis carinii, tuberculosis, toxoplasmosis, aspergillosis, zoster infection, legionella pneumophilia, etc.) were recorded. BK infections were not recorded.

Diagnosis of CMV disease required the presence of viral replication and clinical symptoms.

Diagnosis was blinded from biological evaluation. Most centres performed weekly monitoring until months 2 and at each visit after the second month.

### Statistical analysis

Arithmetic mean was calculated and expressed as + SD. For normally distributed variables, t test was used for continuous variables and chi-2 test for dichotomous variables. Abnormally distributed variables were either log-transformed or split in tertiles.

Correlations were calculated through Spearman test. Multiple regressions were used to determine factors associated with insulin sensitivity and secretion.

Outcomes were studied in the exploratory cohort. Using log rank tests on Kaplan Meier nonparametric estimates of the survival without outcome distribution in the first year post-transplant, we selected variables with a *p* value lower than, or equal to, 0.20. The selected variables were included into a Cox proportional hazards model, and a backward stepwise selection process was performed, this time at a classical α = 0.05. Results are expressed as hazard ratio (HR) and 95% confidence interval (CI), with a *p* value testing the null hypothesis: HR = 1. Therefore when *p* value is less than 0.05, HR is significantly different from 1, either greater than 1 (i.e., risk of acute rejection is increased) or less than 1 (i.e., risk of acute rejection is decreased). Assumptions of Cox models (log-linearity, proportionality of risk in time) were met in this analysis.

## Results

### Exploratory cohort

#### Demographic characteristics

Demographic and clinical characteristics of the study population are depicted in Table 1. HD and PD patients did not differ for any parameters.

**Table 1:** Demographic and clinical characteristics of the study population (exploratory cohort)

	PD *n* = 138	HD *n* = 537	p
Age (years)	51 ± 15	53 ± 13	0.093
Gender (% male)	75%	68%	0.626
Dialysis duration (months)	33 + 20	63 + 123	0.168
Diabetes (%)	19%	20%	0.775
BMI	25.8 ± 4.9	25.8 ± 4.8	0.988
Pre-transplant CMV exposure	55%	54%	0.822
Induction therapy (% of patients having received ATG)	33%	36%	0.567
Tacrolimus (%)	62%	58%	0.348
MMF (%)	97%	97%	0.960
Scheduled stéroids withdrawal (%)	8%	9%	0.720

Importantly, age and CMV exposure were similar in the two groups.

#### Immune phenotype

**Fig. 1:**
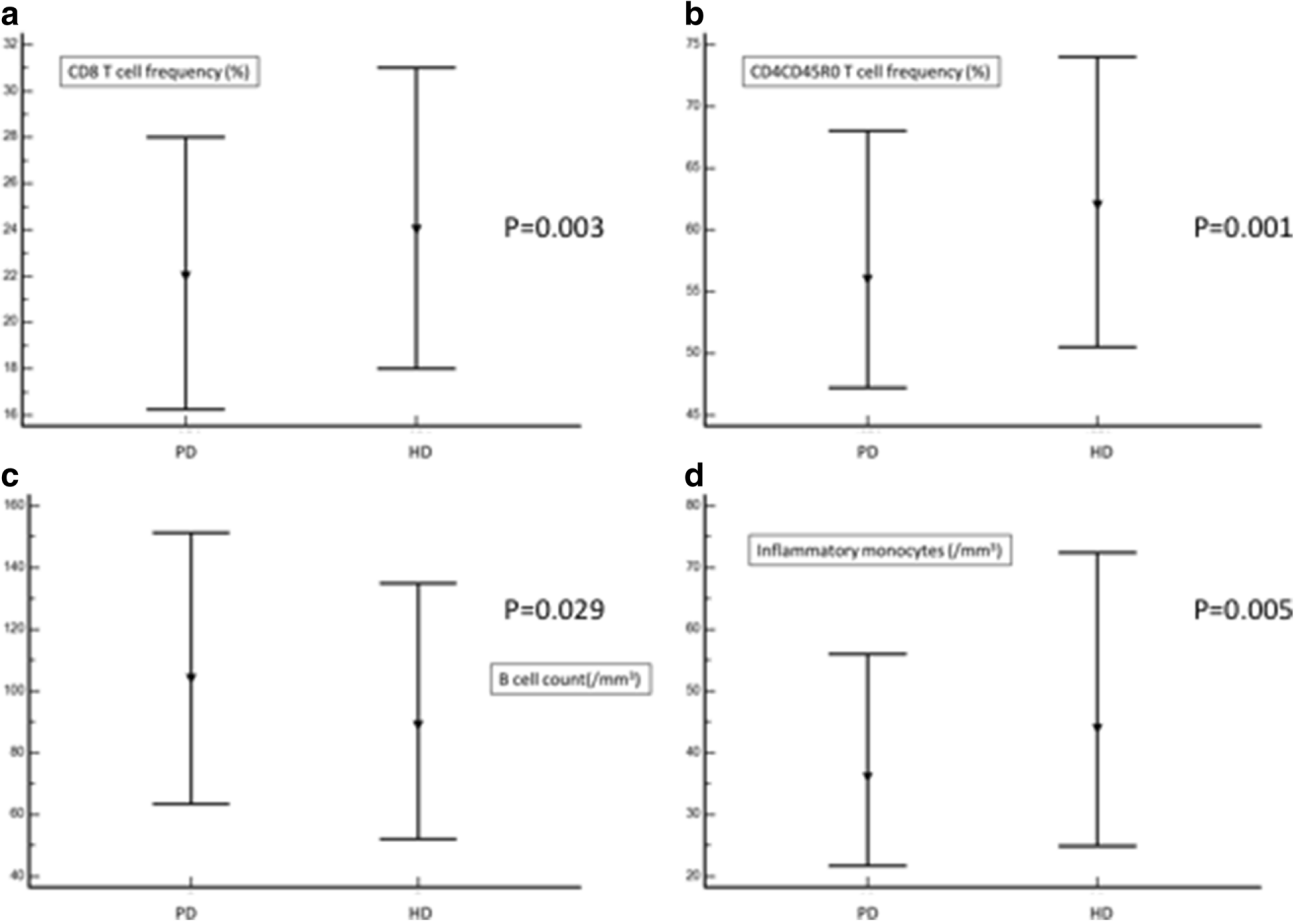
CD8+ T cell frequency (1**a**), CD4 + CD45R0 T cell frequency (1**b**), B cell count (1**c**), and inflammatory monocytes count (1D) in PD and HD patients. Overall, the results suggest more pronounced inflammation (increased number of inflammatory monocytes) and nonspecific features of immune senescence and/or activation (increased number of CD8+ T cells and CD4 + CD45R0 T cells, and decreased number of B cells) in HD patients compared to PD patients

Patients on HD exhibited higher frequency of both CD4 + CD45RO+ (62% (15–97) vs 56% (23–95); *p* = 0.001) (Fig. 1a) and CD8+ (24% (7–61) vs 22% (8–42); *p* = 0.003) T cells (Fig. 1b). Other T cell subsets number and frequencies were similar between PD and HD patients.

We separately studied the association between CD8+ T cell frequency and dialysis modality in CMV-exposed and CMV-naïve patients. HD was marginally associated with higher CD8+ T cell frequency in CMV-naïve (OR, 1.71 95%CI [0.92–3.17], *p* = 0.084), but strongly in CMV-exposed (OR, 1.97 95%CI [1.19–3.28], *p* = 0.009) patients.

HD patients had reduced CD4/CD8 ratio as compared with PD patients (2.36 + 1.36 vs 2.81 + 1.45; *p* = 0.007).

CD4 + CD25 + FoxP3+ Treg were similar in the two groups (3.6 + 2.2% vs 3.4 + 3.2%, in HD and DP patients respectively; *p* = 0.814).

NK cell count (139/mm^3^ (4–1404) vs 3144/mm^3^ (12–999); *p* = 0.328) did not differ between the two groups of patients.

Although B cell frequency was similar in the two groups, total B cell count was higher in PD patients (104/mm^3^ (87–116) vs 89/mm^3^ (81–96); *p* = 0.029) (Fig. 1c).

Total monocytes count was similar in the two groups of patients. Nevertheless, those on HD had higher inflammatory monocytes counts compared to patients on PD (44/mm^3^ (1–520) vs 36/mm^3^ (1–161); *p* = 0.005) (Fig. 1d). CRP levels did not differ between the two groups.

#### Outcomes

##### Acute rejection

119 patients (17.6%) experienced acute rejection.

In univariate analysis, PD was marginally associated with acute rejection (HR, 1.47; 95%CI, 0.93 to 2.33; *p* = 0.109). The rate of acute rejection was 21.5 and 17.6% in HD and PD patients, respectively (Table 2).

**Table 2:** Incidence of post-transplant outcomes according to pre-transplant dialysis modality

	PD *n* = 138	HD *n* = 537	p
Acute rejection	17,6%	21,5%	0.109
CMV disease	18.2%	17%	0.745
Opportunistic infection	23.1%	27.2%	0.342
Severe bacterial infection	34.1%	35.4%	0.771

In multivariate Cox model, both delayed graft function (HR, 1.84; 95%CI, 1.19 to 2.84; *p* = 0.006) and PD (HR, 1.61; 95%CI, 1.02 to 2.56; *p* = 0.041) were associated with acute rejection.

Age was not associated with acute rejection.

##### Infection

We did not observe any differences in the incidence of infectious complications between HD and PD patients (Table 2).

### Cognitive cohort

#### Demographic characteristics

Demographic and clinical characteristics of the study population are depicted in Table 3. HD and PD patients did not differ for any parameters.

**Table 3:** Demographic and clinical characteristics of the study population (cognitive cohort)

	PD *n* = 37	HD *n* = 47	p
Age (years)	67 ± 19	68 ± 14	0.770
Gender (% male)	75%	68%	0.626
Dialysis duration (months)	33 ± 20	63 ± 123	0.168
Diabetes (%)	36	34	0.900
BMI	27.1 ± 4.7	26.7 ± 4.7	0.703
25OH D3 (ng/ml)	24 + 9	30 + 11	0.018
PTH (pg/ml)	393 + 313	401 + 253	0.961
Ferritin (ng/ml)	277 ± 277	481 ± 396	0.007
Albumin (g/l)	32 ± 4	34 ± 3	0.049

Importantly, age and CMV exposure were similar in the two groups.

Ferritin levels markedly differ between HD and PD patients (481 ± 396 vs 277 ± 277 ng/ml; *p* = 0.007).

#### Immune phenotype

##### Inflammation

CRP levels did not significantly differ between PD and HD patients.

##### CD4

Both CD4 T cell count and frequency were similar in HD and PD patients.

**Fig. 2:**
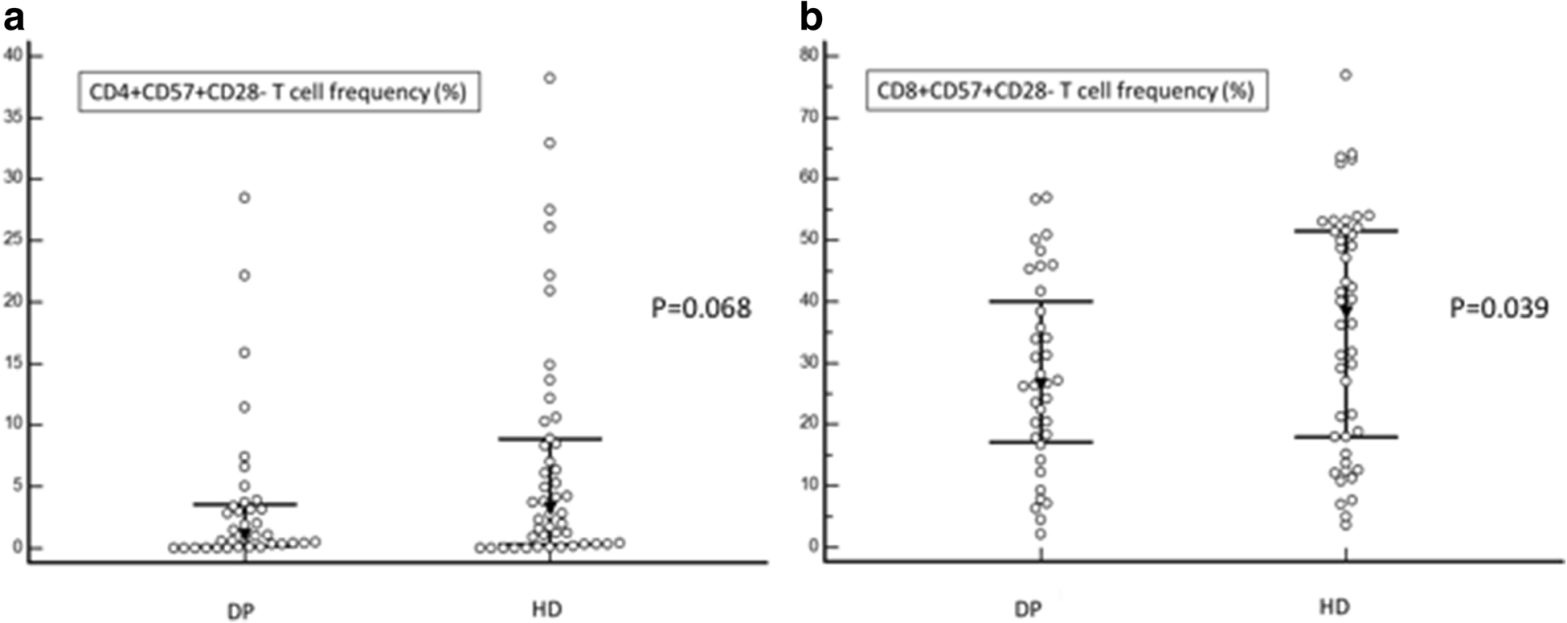
CD4 + CD57 + CD28- (2A) and CD8 + CD57 + CD28- (2B) T cell frequencies in PD and HD patients. Both CD4 + CD57 + CD28- and CD8 + CD57 + CD28- T cell frequencies are increased in HD patients suggesting enhanced replicative senescence

Nevertheless, CD4 + CD57 + CD28- T cell frequency was marginally higher in HD patients (3.25% (0–38.2) vs 1.05% (0–28.5); *p* = 0.068) (Fig. 2a). CMV exposure was the major determinant of CD4 + CD57 + CD28- T cell frequency (*p* < 0.001). Whereas haemodialysis was associated with higher CD4 + CD57 + CD28- T cell frequency in CMV-exposed patients (OR, 3.27 95% CI [1.01–10.62], *p* = 0.048), such a difference between dialysis modalities w as not observed in CMV-naïve patients (OR, 3.00 95%CI [0.28–32.46], *p* = 0.366). There was a trend towards a correlation between dialysis duration and CD4 + CD57 + CD28- T cell frequency (*r* = 0.20; *p* = 0.066).

Both hemodialysis (OR, 3.06 95%CI [0.95–9.83], *p* = 0.061) and CMV exposure (OR, 5.05 95%CI [1.31–19.43], *p* = 0.018) predicted high frequency of CD4 + CD57 + CD28- T cells.

##### CD8

Patients on HD exhibited higher frequency of CD8+ T cells (33% (11–60) vs 24% (11–55); *p* = 0.012) (Fig. 2b). Both CD8 + CD57 + CD28- T cell count (110/mm^3^ (52–173) vs 57/mm^3^ (29–61); *p* = 0.034) and frequency (38.5% (3.6–76.8) vs 26.1 (2.1–46.9); *p* = 0.039) were higher in HD patients. The difference was mainly due to higher TEMRA cells in CMV-exposed patients on HD compared to those on PD. Indeed, HD was strongly associated with higher TEMRA frequency in CMV-exposed patients (OR, 3.70 95%CI [1.14–13.18], *p* = 0.023). This association was not observed in CMV-naïve patients (OR, 0.59 95% CI [0.11–3.20], *p* = 0.540). The effect was independent of age. The difference was not significant in CMV-naïve patients.

Neither CD8+ T cell frequency (*r* = 0.05; *p* = 0.686) nor CD8 + CD57 + CD28- T cell frequency (*r* = − 0.02; *p* = 0.914) were related to dialysis duration.

CD4/CD8 ratio was higher in PD patients (3.3 ± 1.7 vs 2.6 ± 1.8; *p* = 0.015).

##### TREC

TREC numbers did not differ between HD and PD patients.

##### Telomere length and telomerase activity

Telomere length did not differ according to dialysis modality. Nevertheless, we observed a trend towards higher telomerase activity in PD patients (11 ± 13 vs 6 ± 11; *p* = 0.053).

### Iron overload

Because ferritin levels markedly differed between PD and HD patients and because intravenous iron administration, more widely used in HD patients, may induce oxidative stress, a supposed trigger of ESRD-induced immune senescence, we studied whether iron overload may explain at least in part our result.

Iron overload was defined by ferritin levels above 290 ng/ml [12].

TEMRA counts and frequencies were similar in patients with or without iron overload.

**Fig. 3:**
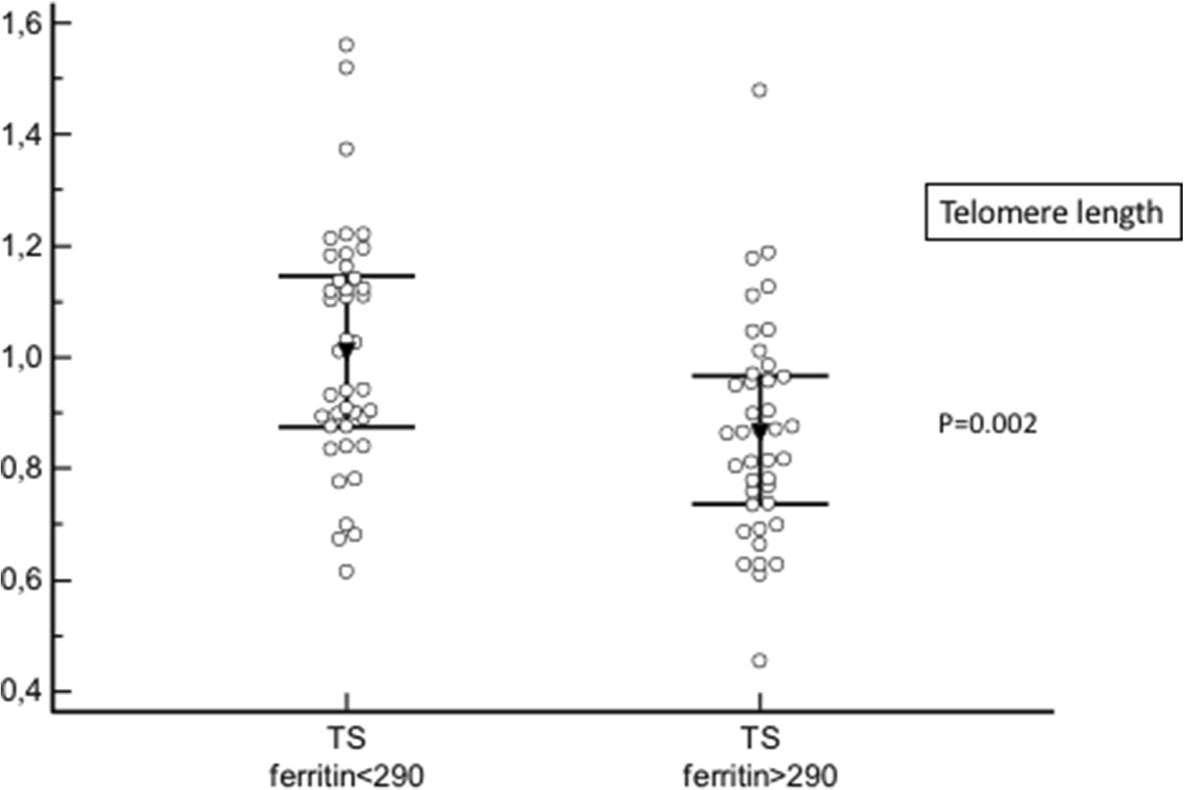
Telomere length according to iron status. Telomere length is reduced in patients with iron overload

**Fig. 4:**
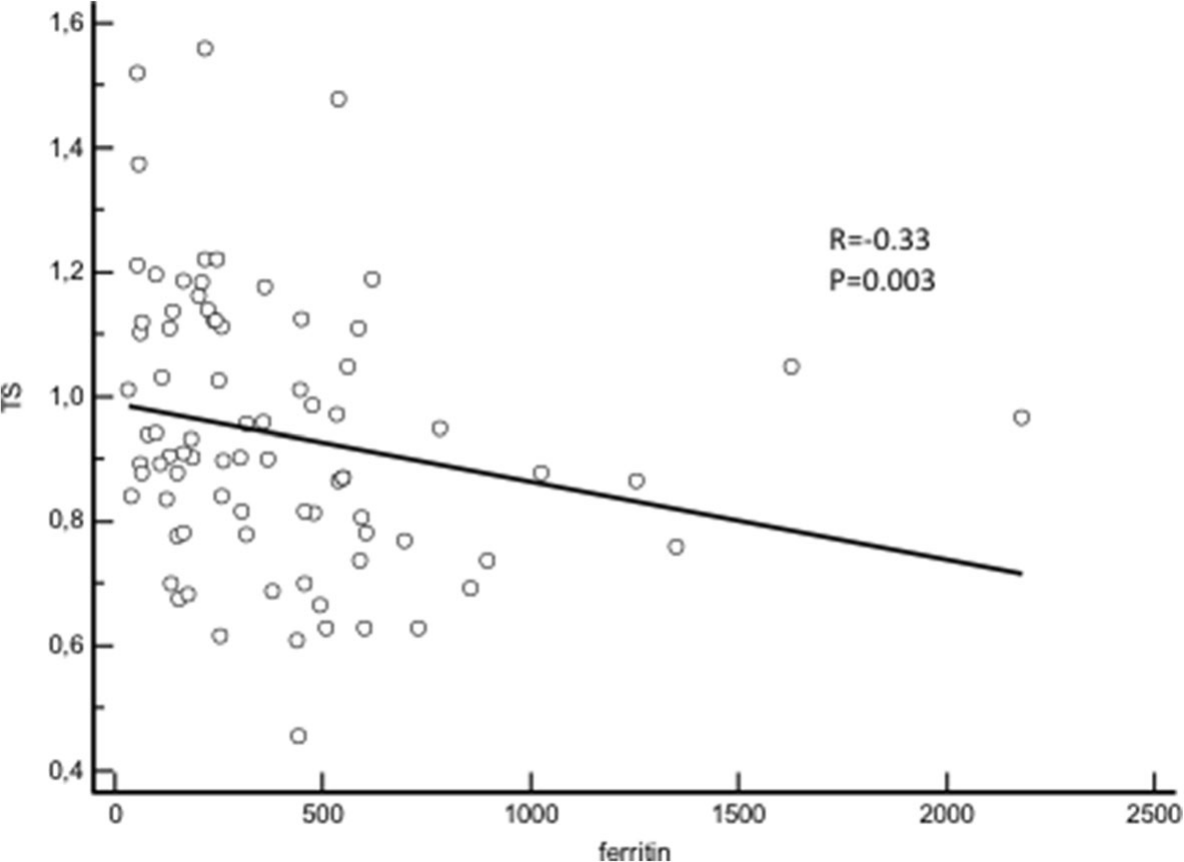
Relationship between ferritin concentrations and telomere length. Telomere length correlates with iron status determined by ferritin levels

By contrast, telomere length was shorter in those with iron overload (0.86 (0.45–1.48) vs 1.01 (0.61–1.56); *p* = 0.002) (Fig. 3). Ferritin levels and telomere length were closely related (*r* = − 0.33; *p* = 0.003) (Fig. 4).

In logistic regression, high ferritin level predicted low telomere length (OR, 5.56 95% CI [1.78–16.67], p = 0.002, for telomere length < 0.87 (median value)). The association persisted after adjustment for age (OR, 4.76 95% CI [1.81–12.50], *p* = 0.023).

## Discussion

Our study reports different immune profile according to dialysis modality. Hemodialysis is associated with more sustained inflammation and lymphocyte activation/exhaustion. The differential immune profile may contribute to an increased incidence of acute rejection in patients on PD before transplantation. By contrast, HD-related accelerated aging may favour infections and ESRD-related cardiovascular disease.

Peritonitis, bio-incompatible solutions, and peritoneal catheter may induce inflammation and T cell activation and aging. Nevertheless, whether PD aggravates systemic ESRD-related inflammation is unclear. Whereas some studies reported higher IL-6 concentrations with longer PD duration [13, 14], others did not observe any burden in IL-6 or CRP levels [15]. Several studies demonstrated inflammation burst with HD procedure [16–18]. Moreover, whereas TLR2 and TLR4 are over-expressed on monocytes from patients on haemodialysis [19], the expression of TLR4 has been reported to be reduced on monocytes in patients with chronic kidney disease (CKD) not receiving dialysis [20]. This suggests that intermittent activation of monocytes by the dialyzer might result in up-regulation of TLR4. Accordingly, we observed both higher inflammatory monocytes counts in patients on HD as compared to those on PD. We hypothesized that sustained low-grade inflammation may contribute to immune responses to self-antigens and pathological aging by promoting stem cell exhaustion [21]. Alternatively, sustained repeated antigenic stimulation of T cells during hemodialysis procedure may cause more intense proliferation and accelerated aging compared to PD.

It is reported that in cell types with high proliferative capacity, telomerase may be induced in response to different signal to maintain telomere length and protect chromosomes against damage [22–25]. Nevertheless, we observed higher telomerase activity in patients on PD as compared to those on HD. This result suggests a possible inhibition of telomerase activity. Indeed, certain cytokines secreted during hemodialysis session, such as IFN-α, may inhibit telomerase activity in hematopoietic cells [26, 27].

ESRD-induced T cell exhaustion may play a relevant role in accelerated atherosclerosis observed in dialysis patients. Both CD4 + CD28- and CD8 + CD28- T cells may promote endothelial cell damage, inflammation and destabilization of atherosclerotic plaques, and arterial calcification. Such T cell populations have been previously associated with cardiovascular outcomes in HIV-infected patients [28]. More recently, we reported that CD8 + CD28-CD57+ T cell number was associated with major cardiovascular outcomes in dialysis patients [5]. CD4 + CD28- T cell expansion was previously reported in ESRD patients and strongly associated with CMV seropositivity [4]. Moreover, CMV-driven expansion of CD28- T lymphocytes may explain the association between CMV seropositivity and atherosclerotic events in both dialysis [29] and kidney transplant patients [30]. We also observed marked TEMRA expansion in CMV-exposed patients. The large majority of CD28- T cells are likely to be CMV-specific and to only proliferate in responses to CMV. This would suggest more frequent CMV reactivation in HD patients compared to PD patients. Otherwise, the differential effect of haemodialysis in CMV-exposed and CMV-naïve patients suggests that haemodialysis procedures may amplify rather than generate the TEMRA pool. Alternatively, some cytokines, such interferon-α, may accelerate the loss of CD28- on T cells as well as inhibit telomerase activity in the absence of CMV antigenic challenge [27, 31].

Previous studies reported that the degree of ESRD-related T cell dysfunction may affect the incidence of acute rejection after kidney transplantation. Betjes et al. showed that patients with higher frequency of terminally differentiated CD8+ TEMRA cells had a decreased risk of acute rejection [32]. Increased CD8+ TEMRA cells number is associated with reduced T cell diversity that may result in reduced diversity of alloreactive T cells [33–35]. Alternatively, these cells may have suppressive effects by reducing the efficacy of antigen-presenting cells to induce T- cell proliferation. Our group reported a tendency for high late stage differentiated CD4 T cell frequency at transplant to be associated with acute rejection in ATG-treated patients [11]. Similar findings have been reported in liver transplantation [36]. By contrast, PD before transplantation has not been previously associated with an increased risk of acute rejection [37]. Delayed graft function is likely to be more frequently reported in HD patients than in PD patients because of better residual renal function in those in PD. This may have masked the association between PD and acute rejection. By contrast, HD was not associated with an increased incidence of post-transplant infections. However, previous studies suggest pre-transplant TEMRA frequency may mitigate the risk of post-transplant infections. Indeed, we previously reported that immune risk profile is associated with post-transplant infectious outcomes [38]. The absence of association may result from confounding parameters (including short duration of follow-up, consideration of different kinds of infections, immunosuppressive drugs, …) or alternatively from a differential impact of TEMRA on allo-immune and anti-infectious responses.

Ferritin levels were higher in HD patients. Many studies reported better iron supplementation in HD patients compared to PD patients [39]. Intravenous iron administration induces oxidative stress [40]. Nevertheless, we did not observe differences in TEMRA counts or frequencies according to iron status. By contrast, iron overload was associated with shorter telomere length. Other studies reported associations between iron overload and telomere length [41–43]. We previously reported reduced telomere length to be associated with mortality in dialysis patients [5]. Excessive iron load also contributes to ferroptosis [44]. Ferroptosis has an important role in sterile inflammatory conditions such as tissue acute injury, ischemic-reperfusion injury, and neurotoxicity. Further studies should determine whether iron-induced oxidative stress may contribute to morbidity and mortality in dialysis patients.

Association does not preclude causality. As a consequence, we cannot assume a selection bias in the choice of dialysis modality resulting in different immune profiles. Nevertheless, the absence of relevant clinical differences between HD and PD patients make this very unlikely. In fact, this comparison does not suffer usual bias of studies comparing PD and HD patients because we compared a more homogenous subgroup of patients listed on the transplant waiting list. Consequently, major differences between patients are not attempted. Because of the cross-sectional nature of the study, we cannot analyse changes in immunological parameters after and during renal replacement therapy. Nevertheless, we included a very large population in the exploratory cohort and we confirmed primary results in the cognitive cohort.

## Conclusions

Our study provides new data concerning ESRD-associated immune senescence. We analysed whether dialysis modality may interfere with immune status. We observed accelerated aging of the immune system in hemodialysis patients as compared with those on peritoneal dialysis. The increase in terminally differentiated CD4^+^ and CD8^+^ T cells may explain a lower risk of acute kidney rejection in patients previously on HD. Finally, we reported that iron overload correlates with shorter telomere length. Telomere shortening has been associated with excess in mortality in dialysis patients. This result clearly raises the question of the current iron use in dialysis patients.

## Abbreviations

CKD: Chronic kidney disease
CMV: Cytomegalovirus
eGFR: Estimated glomerular filtration rate
ESRD: End-stage renal disease
HD: Hemodialysis
MDRD: Modified diet in renal disease
PD: Peritoneal dialysis
RTA: Relative telomerase activity
RTL: Relative telomere length
RTR: Renal transplant recipients
TEMRA: Terminally effector memory

## References

[1] Sakhuja A, Nanchal RS, Gupta S, Amer H, Kumar G, Albright RC, Kashani KB (2016). Trends and outcomes of severe sepsis inpatients on maintenance dialysis. Am J Nephrol.

[2] Stewart JH, Vajdic CM, van Leeuwen MT, Amin J, Webster AC, Chapman JR, McDonald SP, Grulich AE, McCredie MR (2009). The pattern of excess cancer in dialysis and transplantation. Nephrol Dial Transplant.

[3] Soni R, Horowitz B, Unruh M (2013). Immunization in end-stage renal disease: opportunity to improve outcomes. Semin Dial.

[4] Betjes MGH, Huisman M, Weimar W, Litjens NHR (2008). Expansion of cytolytic CD4+CD28- T cells in end-stage renal disease. Kidney Int.

[5] Crepin T, Legendre M, Courivaud C, Rebibou JM, Ferrand C, Laheurte C, Vauchy C, Gaiffe E, Saas P, Ducloux D, Bamoulid J. Uremia-induced immune senescence and clinical outcomes in chronic disease patients. Nephrol Dial Transplant 2018 (*in press*). 10.1093/ndt/gfy276 30202981

[6] Betjes MG, Meijers RW, Litjens NH (2013). Loss of renal function causes premature aging of the immune system. Blood Purif.

[7] Betjes MG (2013). Immune cell dysfunction and inflammation in end-stage renal disease. Nat Rev Nephrol.

[8] Meijers RWH, Litjens NHR, de Wit EA, Langerak AW, van der Spek A, Baan CC, Weimar W, Betjes MGH (2012). Uremia causes premature ageing of the T-cell compartment in end-stage renal disease patients. Immun Ageing.

[9] Bamoulid J, Courivaud C, Crepin T, Carron C, Gaiffe E, Roubiou C, Laheurte C, Moulin B, Frimat L, Rieu P, Mousson C, Durrbach A, Heng A-E, Rebibou JM, Saas P, Ducloux D (2016). Pre-transplant thymic function predicts acute rejection in ATG-treated renal transplant recipients. Kidney Int.

[10] Ducloux D, Courivaud C, Bamoulid J, Vivet B, Chabroux A, Deschamps M, Rebibou JM, Ferrand C, Chalopin JM, Tiberghien P, Saas P (2010). Prolonged CD4 T cell lymphopenia increases morbidity and mortality after renal transplantation. J Am Soc Nephrol.

[11] Crepin T, Carron C, Roubiou C, Gaugler B, Gaiffe E, Simula-Faivre D, Ferrand C, Tiberghien P, Chalopin J-M, Moulin B, Frimat L, Rieu P, Saas P, Ducloux D, Bamoulid J (2015). ATG-induced accelerated immune senescence: clinical implications in renal transplant recipients. Am J Transplant.

[12] Rostoker G, Griuncelli M, Loridon C, Magna T, Machado G, Drahi G, Dahan H, Janklewicz P, Cohen Y. Reassessment of iron biomarkers for prediction of dialysis iron overload: an MRI study. PLoS One. 2015:e0132006. 10.1371/journal.pone.0132006 PMC4504469 26182077

[13] Lambie M, Chess J, Donovan L, Kim YL, Do JY, Lee HB, Noh H, Williams PF, Williams AJ, Davison S, Dorval M, Summers A, Williams JD, Bankart J, Davies SJ, Topley N (2013). Independent effects of systemic and peritoneal inflammation on peritoneal dialysis survival. J Am Soc Nephrol.

[14] Pecoits-Filho R, Carvalho MJ, Stenvinkel P, Lindholm B, Heimbürger O (2006). Systemic and intraperitoneal interleukin-6 system during the first year of peritoneal dialysis. Perit Dial Int.

[15] Cho JH, Hur IK, Kim CD, Park SH, Ryu HM, Yook JM, Choi JY, Choi HJ, Choi HJ, Park JW, Do JY, Kim YL (2010). Impact of systemic and local peritoneal inflammation on peritoneal solute transport rate in new peritoneal dialysis patients: a 1-year prospective study. Nephrol Dial Transplant.

[16] Oldani S, Finazzi S, Botazzi B, Garlanda C, Baldassarre E, Valaperta S, Cuccovillo I, Albino M, Child M, Montanelli A, Graziani G, Badalamenti S (2012). Plasma pentraxin-3 as a marker of biocompatibility in hemodialysis patients. J Nephrol.

[17] Yamamoto T, Nascimento MM, Hayashi SY, Qureshi AR, Waniewski J, Brodin LA, Anderstam B, Lind B, Riella MC, Seeberger A, Lindholm B (2013). Changes in circulating biomarkers during a single hemodialysis session. Hemodialysis Int.

[18] Bitla AR, Reddy PE, Manohar SM, Vishnubhotla SV, Pemmaraju Venkata Lakshmi Narasimha SR (2010). Effect of a single hemodialysis session on inflammatory markers. Hemodialysis Int.

[19] Gollapudi P, Yoon JW, Gollapudi S, Pahl MV, Vaziri ND (2010). Leukocyte toll-like receptor expression in end-stage kidney disease. Am J Nephrol.

[20] Ando M, Shibuya A, Tsuchiya K, Akiba T, Nitta K (2006). Reduced expression of toll-like receptor 4 contributes to impaired cytokine response of monocytes in uremic patients. Kidney Int.

[21] Lopez-Otin C, Blasco MA, Partridge L, Serrano M, Kroemer G (2013). The hallmarks of aging. Cell.

[22] Chiu CP, Dragowska W, Kim NW, Vaziri H, Yui J, Thomas TE (1996). Differential expression of telomerase activity in hematopoietic progen-itors from adult human bone marrow. Stem Cells.

[23] Engelhardt M, Kumar R, Albanell J, Pettengell R, Han W, Moore MA (1997). Telomerase regulation, cell cycle, and telomere stability in primitive hemato-poietic cells. Blood.

[24] Plunkett FJ, Franzese O, Finney HM, Fletcher JM, Belaramani LL, Salmon M (2007). The loss of telomerase activity in highly differentiated CD8+CD28-CD27- T cells is associated with decreased Akt (Ser473) phos-phorylation. J Immunol.

[25] Akbar AN, Vukmanovic-Stejic M (2007). Telomerase in T lymphocytes: use it and lose it?. J Immunol.

[26] Xu D, Erickson S, Szeps M, Gruber A, Sangfelt O, Einhorn S (2000). Interferon alpha down-regulates telomerase reverse transcriptase and telomerase activity in human malignant and nonmalignant hematopoietic cells. Blood.

[27] Reed, J. R., M. Vukmanovic-Stejic, J. M. Fletcher, M. V. Soares, J. E. Cook, C. H. Orteu, S. E. Jackson, K. E. Birch, G. R. Foster, M. Salmon, et al. 2004. Telomere erosion in memory T cells induced by telomerase inhibition at the site of antigenic challenge in vivo. J Exp Med 2004:199; 1433–1443. 10.1084/jem.20040178 PMC2211820 15148341

[28] Kaplan RC, Sinclair E, Landay AL (2011). T cell activation predicts carotid artery stiffness in HIV-infected women. Atherosclerosis.

[29] Betjes MG, Litjens NH, Zietse R (2007). Seropositivity for cytomegalovirus in patients with end-stage renal disease is strongly associated with atherosclerotic disease. Nephrol Dial Transplant.

[30] Courivaud C, Bamoulid J, Chalopin JM, Gaiffe E, Tiberghien P, Saas P, Ducloux D (2013). Cytomegalovirus exposure and cardiovascular disease in kidney transplant recipients. J Infect Dis.

[31] Borthwick NJ, Lowdell M, Salmon M, Akbar AN (2000). Loss of CD28 expression on CD8 T cells is induced by IL-2 receptor _ chain signalling cytokines and type I IFN, and increases susceptibility to activation-induced apoptosis. Int Immunol.

[32] Betjes MGH, Meijers RWJ, de Wit EA, Weimar W, Litjens NHR (2012). Terminally differentiated CD8+ Temra cells are associated with the risk of acute kidney allograft rejection. Transplantation.

[33] Franceschi C, Bonafe M, Valensin S (2000). Human immunosenescence: the prevailing of innate immunity, the failing of clonotypic immunity, and the filling of immunological space. Vaccine.

[34] Tulunay A, Yavuz S, Direskenell H, Eksioglu-Demiralp E (2008). CD8+CD28-, suppressive T cells in systemic lupus erythematosus. Lupus.

[35] Cortesini R, LeMaoult J, Ciubotariu R (2001). CD8^+^CD28^j^ T suppressor cells and the induction of antigen-specific, antigen- presenting cellYmediated suppression of Th reactivity. Immunol Rev.

[36] Gerlach UA, Vogt K, Schlickeiser S, Meisel C, Streitz M, Kunkel D, Appelt C, Ahrlich S, Lachmann N, Neuhaus P, Pascher A, Sawitzki B. Elevation of CD4+ differentiated memory T cells is associated with acute cellular and antibody-mediated rejection after liver transplantation. Transplantation 2013; 27–1512–1520. 10.1097/TP.0b013e318290de18 23619734

[37] Tang M, Li T, Liu H (2016). A comparison of transplant outcomes in peritoneal and hemodialysis patients: a meta-analysis. Blood Purif.

[38] Crepin T, Gaiffe E, Courivaud C, Roubiou C, Laheurte C, Moulin B, Frimat L, Rieu P, Mousson C, Durrbach A, Heng A-E, Saas P, Bamoulid J, Ducloux D (2016). Pre-transplant end-stage renal disease-related immune risk profile in kidney transplant recipients predicts post-transplant infections. Transplant Inf Dis.

[39] Wetmore JB, Peng Y, Monda KL, Kats AM, Kim DH, Bradbury BD, Collins AJ, Gilbertson DT (2015). Trends in anemia management practices in patients receiving hemodialysis and peritoneal dialysis: a retrospective cohort analysis. Am J Nephrol.

[40] Liakopoulos V, Roumeliotis S, Gomy X, Dounousi E, Mertens PR. Oxidative stress in hemodialysis patients : a review of literature. Oxidative Med Cell Longev 2017; 2017: 3081856. 10.1155/2017/3081856 PMC5613374 29138677

[41] Shin C, Baik I (2017). Transferrin saturation concentrations associated with telomeric ageing: a population-based study. Br J Nutr.

[42] Murillo-Ortiz B, Ramirez Emiliano J, Hernandez Vazquez WI, Martinez-Garza S, Solorio-Meza S, Albarran-Tamayo F, Ramos-Rodriguez E, Benitez-Bribiesca L (2016). Impact of oxidative stress in premature aging and iron overload in hemodialysis patients. Oxidative Med Cell Longev.

[43] Kepinska M, Szyller J, Milnerowicz H (2015). The influence of oxidative stress induced by iron on telomere length. Environ Toxicol Pharmacol.

[44] Xie Y, Hou W, Song X, Yu Y, Huang J, Sun X, Kang R, Tang D (2016). Ferroptosis: process and function. Cell death Diff.

